# Supplementary material for: AGC family kinase 1 participates in trogocytosis but not in phagocytosis in Entamoeba histolytica
Source: Nat Commun. 2017 Jul 17;8:101. doi: 10.1038/s41467-017-00199-y (PMC5524646; doi:10.1038/s41467-017-00199-y)
Supplement: Supplementary file 1 — Supplementary Information [file 41467_2017_199_MOESM1_ESM.pdf]

File Name: Supplementary Information
Description: Supplementary Figures and Supplementary Tables

File Name: Supplementary Movie 1
Description: Live cell imaging showing presence of GFP-EhAGCK1 in pseudopod like structures formed by trophozoites.

File Name: Supplementary Movie 2
Description: Live imaging showing no preferential localisation of GFP-EhAGCK1 during erythrophagocytosis.

File Name: Supplementary Movie 3
Description: Live imaging showing GFP-EhAGCK2 localisation to the erythrophagocytic cups and association with the newly formed phagosome.

File Name: Supplementary Movie 4
Description: Live imaging data showing recruitment of GFP-EhAGCK1 to narrow tunnels formed during the ingestion of live CHO cells.

File Name: Supplementary Movie 5
Description: Imaging data showing recruitment of GFP-EhAGCK2 to the site of trogocytosis of live CHO cells.

File Name: Supplementary Movie 6
Description: Time lapse imaging showing no preferential recruitment of GFP-EhAGCK1 during phagocytosis of pre-killed CHO cells.

File Name: Supplementary Movie 7
Description: Live imaging showing intense recruitment of GFP-EhAGCK2 to the site of phagocytosis of pre-killed CHO cell.

File Name: Peer Review File
Description:

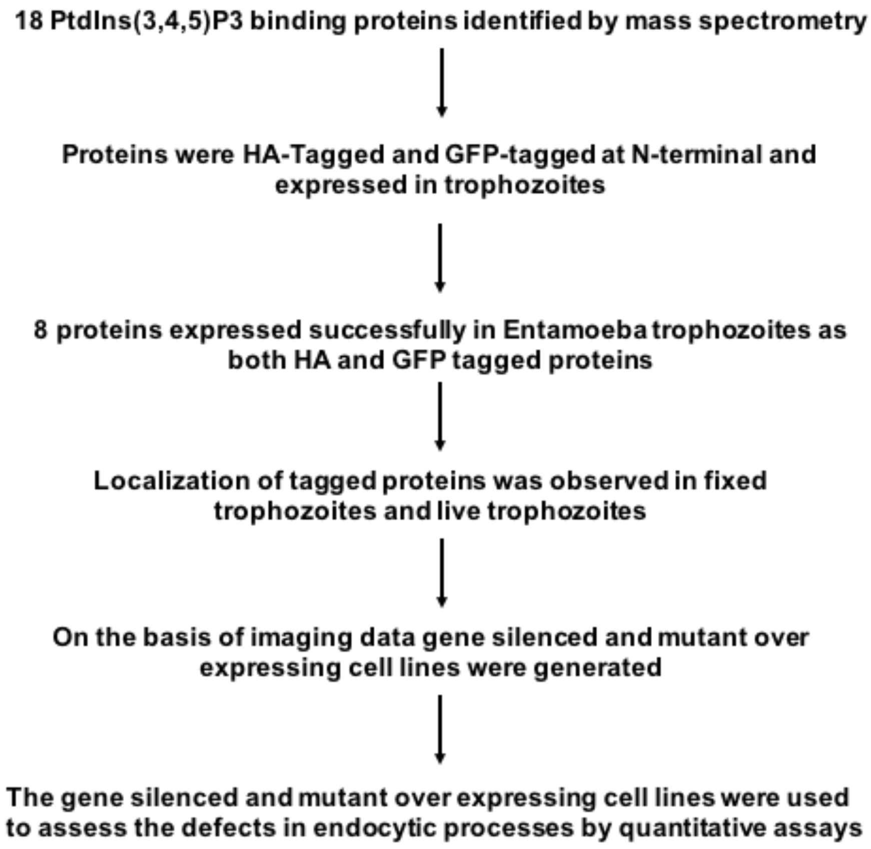

Supplementary Fig. 1. The schematic flow diagram showing the strategy adopted for screening and assigning roles to PtdInsP3 binding proteins in *E. histolytica* biology.

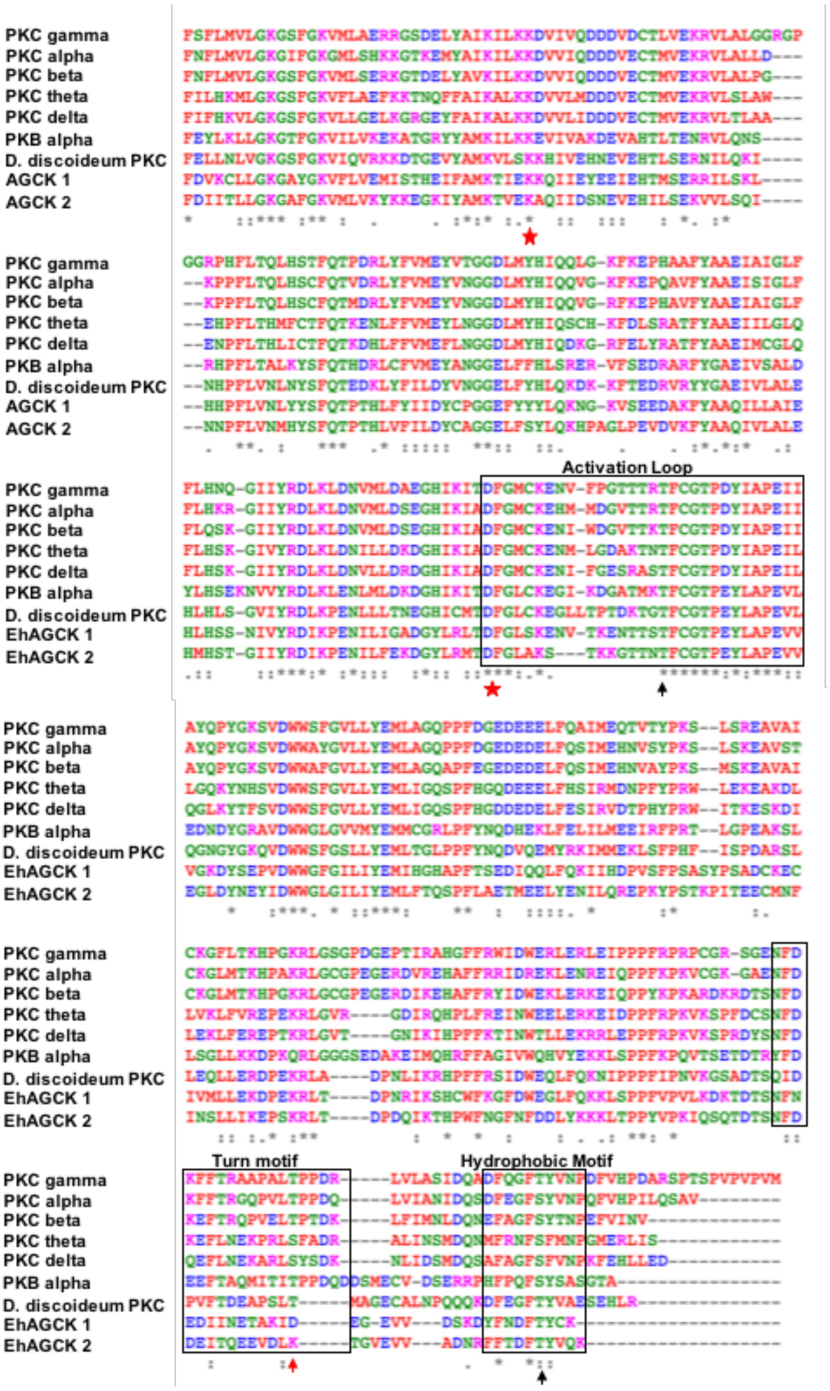

Supplementary Fig. 2. Multiple sequence alignment of kinase domain of EhAGCK1 and EhAGCK2 with similar kinase domain from *Homo sapiens* and *D. discoideum*. The conserved Lys147 and Asp242/241 residues mutated for generating kinase dead mutant has been marked by red star. The activation loop, turn motif and hydrophobic motif are marked by black boundaries. The conserved phosphorylation sites in activation and hydrophobic motif have been marked by black arrow and the missing phosphorylation site in turn motif has been pointed by red arrow.

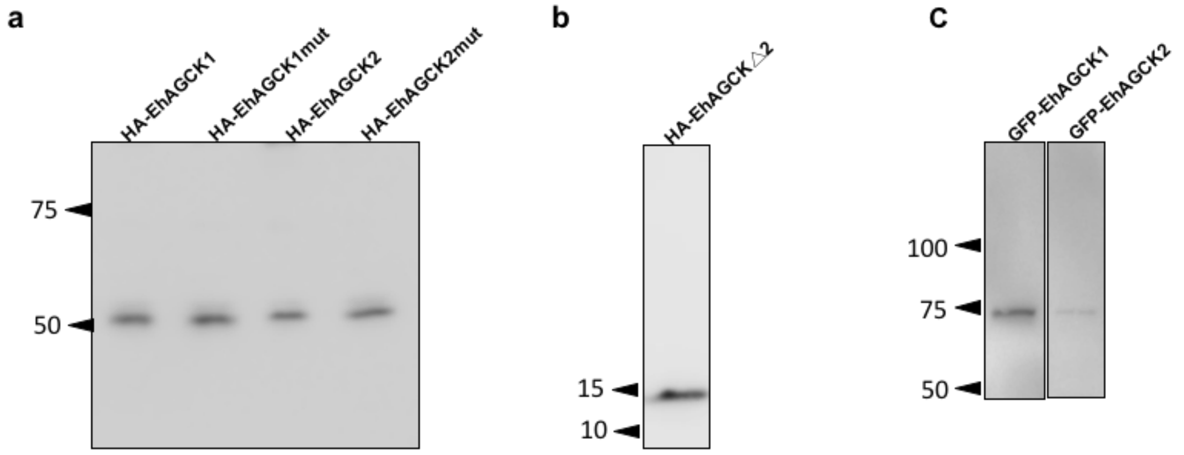

Supplementary Fig. 3. (a) Expression of HA-tagged full length EhAGCK1 and respective kinase dead mutants EhAGCK1mut and EhAGCK2mut in *E. histolytica* trophozoites as detected by anti-HA antiboby. (b) Expression of PH domain of EhAGCK2 (EhAGCKΔ2) in *E. histolytica* trophozoites as detected by anti-HA antibody. (c) Immuno-blot detection of GFP-tagged EhAGCK1 and 2 by anti-GFP monoclonal antibody.

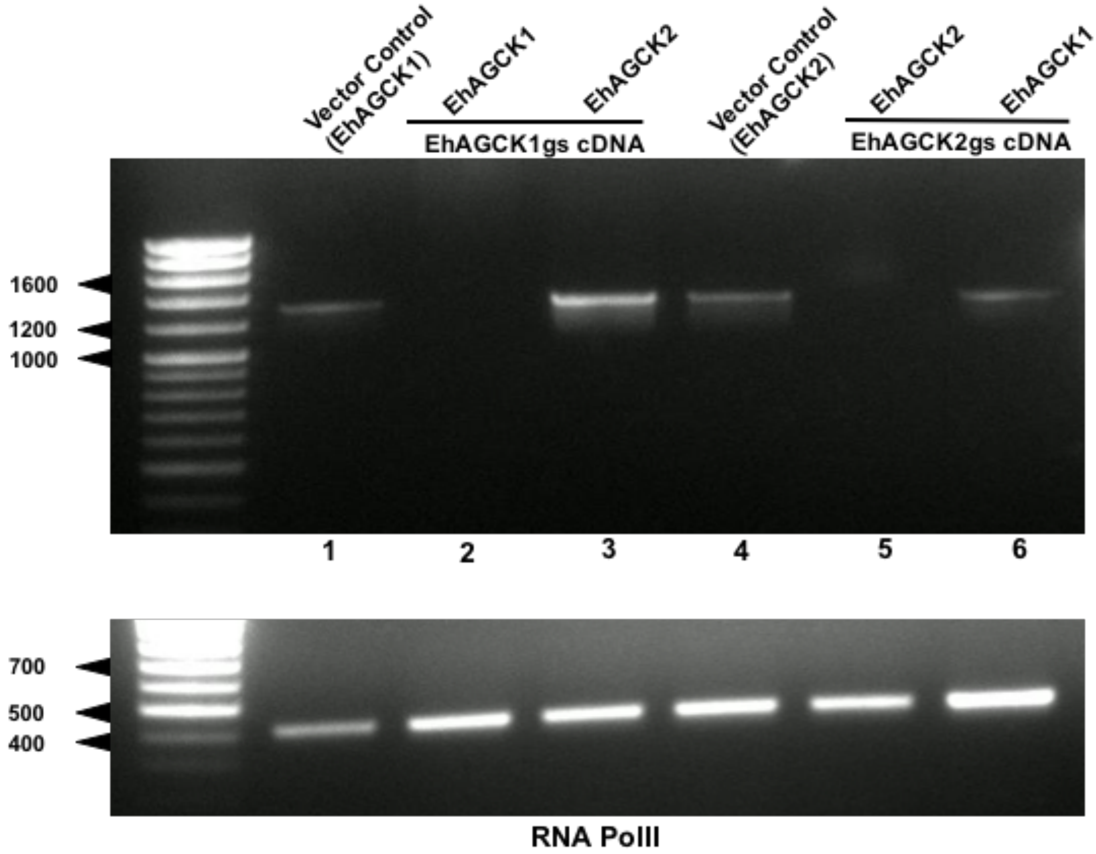

Supplementary Fig. 4. The expression of EhAGCK1 and 2 was silenced by antisense small RNA-mediated transcriptional gene silencing in G3 strain of *E. histolytica*. The expression was monitored by Reverse Transcriptase PCR. Lane 1 is showing expression of EhAGCK1 in vector control cDNA, lane 2 is showing silencing of EhAGCK1 in transfected trophozoites (EhAGCK1gs). Lane 3 is showing expression of EhAGCK2 in EhAGCK1gs cells. Lane 4 is showing expression of EhAGCK2 in vector control cells. Lane 5 showing EhAGCK2 gene silencing in trophozoites (EhAGCK2gs). Lane 6 is showing expression of EhAGCK1 in EhAGCK2gs amoebic cells. The RNA PolII has been used as loading control. The RNA PolII gene was used as loading control. (gs, gene silenced).

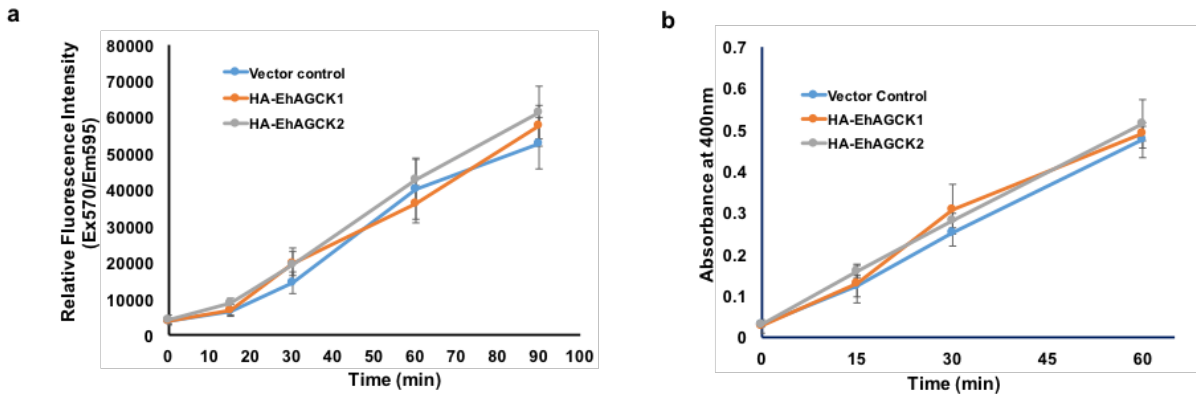

Supplementary Fig. 5. (a) Trophozoites overexpressing wild type HA-EhAGCK1 and 2 showing pinocytosis in time dependent manner. (b) The graph showing erythrophagocytosis by the trophozoites overexpressing wild type protein in time dependent manner. Over expression of EhAGCK1 and 2 does not lead to increase in pinocytic and erythrophagocytic ability of the trophozoites. The experiments were repeated three times independently (N=3) and in triplicates. The error bars represent standard deviation.

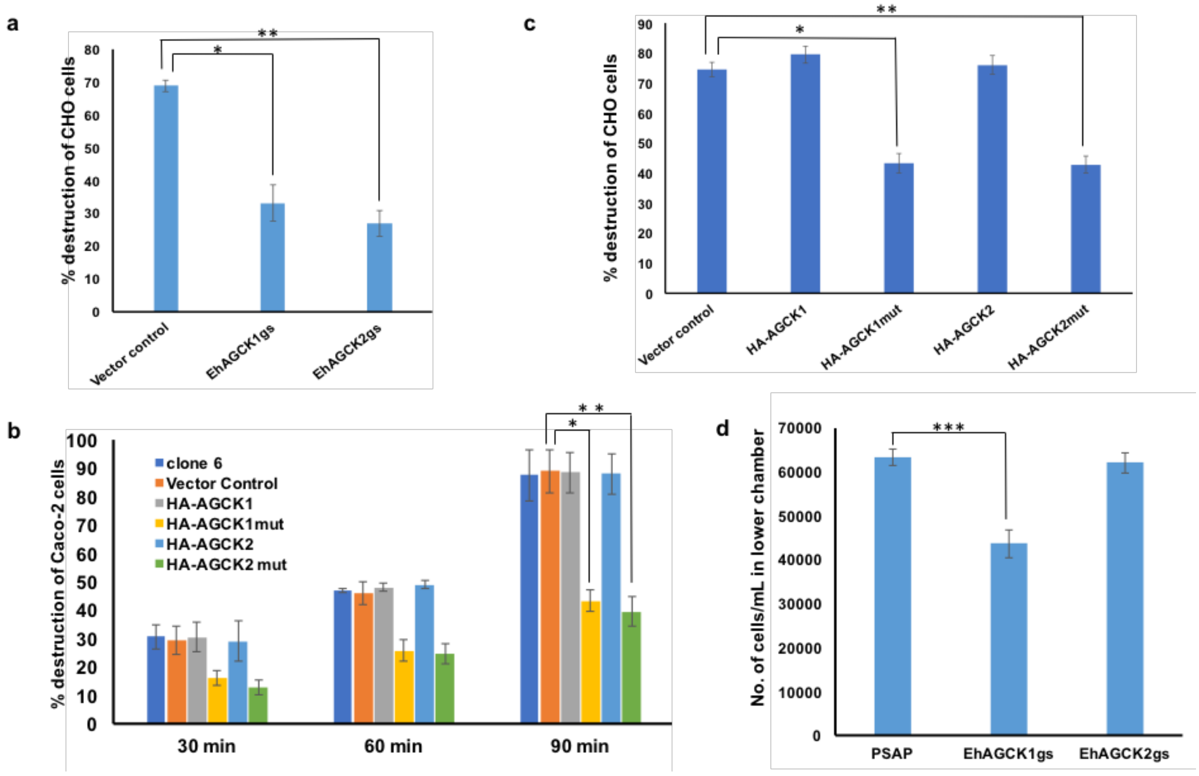

Supplementary Fig. 6. (a) Destruction of monolayer of CHO cells by trophozoites silenced for EhAGCK1 and 2 expression. P value for * is 0.001 and ** is 0.002. (b) *E. histolytica* trophozoites over expressing HA-tagged kinase dead mutants, EhAGCK1mut and EhAGCK2mut exhibit defect in Caco-2 monolayer destruction in time dependent manner. P value for 3 independent experiments for * and ** is 0.002. (c) Overexpression of kinase dead mutants also leads to defect in CHO monolayer destruction. P value for 3 independent experiments for * is 0. 0006 and ** is 0.0005. (d) EhAGCK1gs trophozoites show 20% decrease in transwell migration assay as compared to vector control. P value for the three independent experiments is 0.0001. All the assays were repeated three times independently (N=3) in triplicates. The error bars represent standard deviation. The statistical comparisons are calculated by student t test.

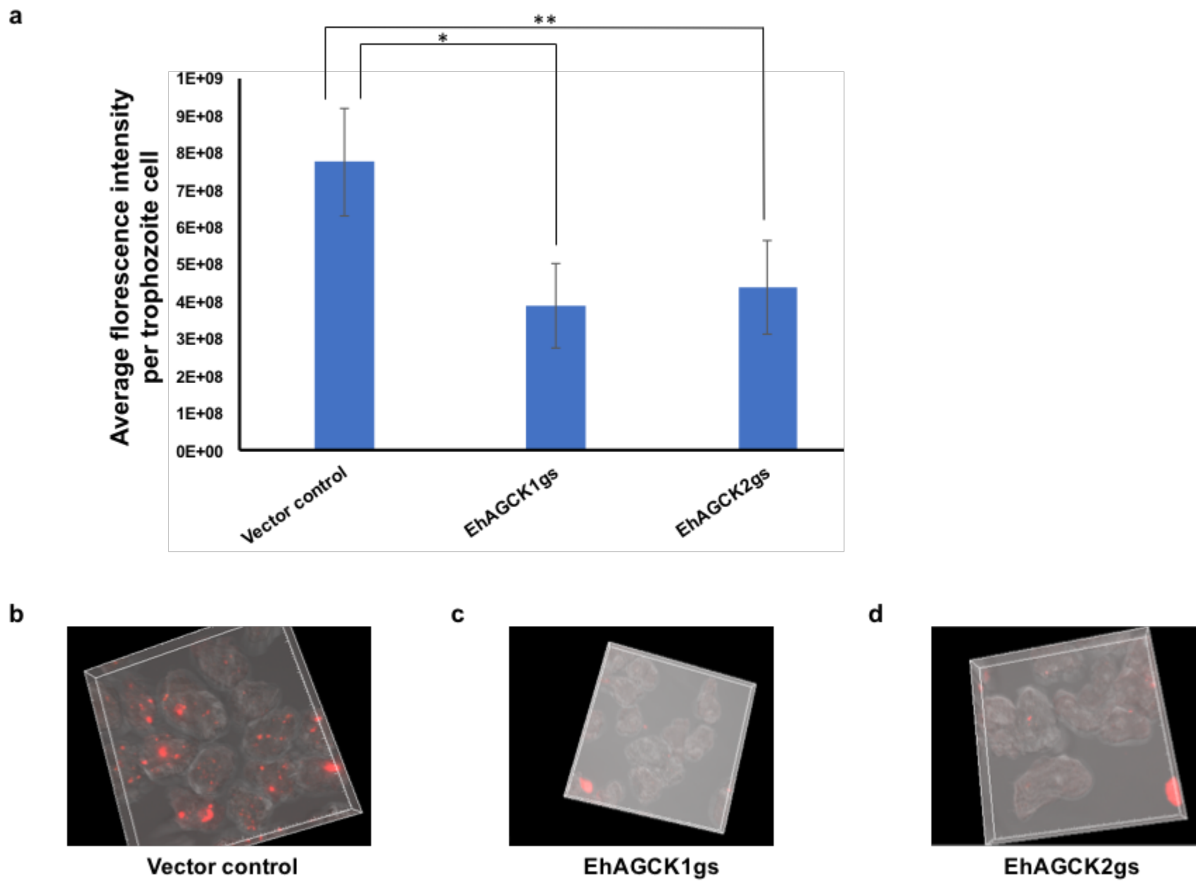

Supplementary Fig. 7. (a) The average fluorescent intensity of labeled CHO cell fragments ingested by Vector control trophozoites, EhAGCK1gs and EhAGCK2gs cells. P value for 3 experiments for * is 0.021 and ** is .037. (b) Representative reconstructed 3-d images of the trophozoites which were selected for analysis by IMARIS 7.6 software. Analysis included only intact and whole amoebic cell in the field. The experiment was carried out three independent times. In every experiment 45 cells per cell line were analyzed. In total 135 cells were analyzed from 3 different experiment per transfected amoebic cell line. The statistical significance of the experiment was calculated by student t test.

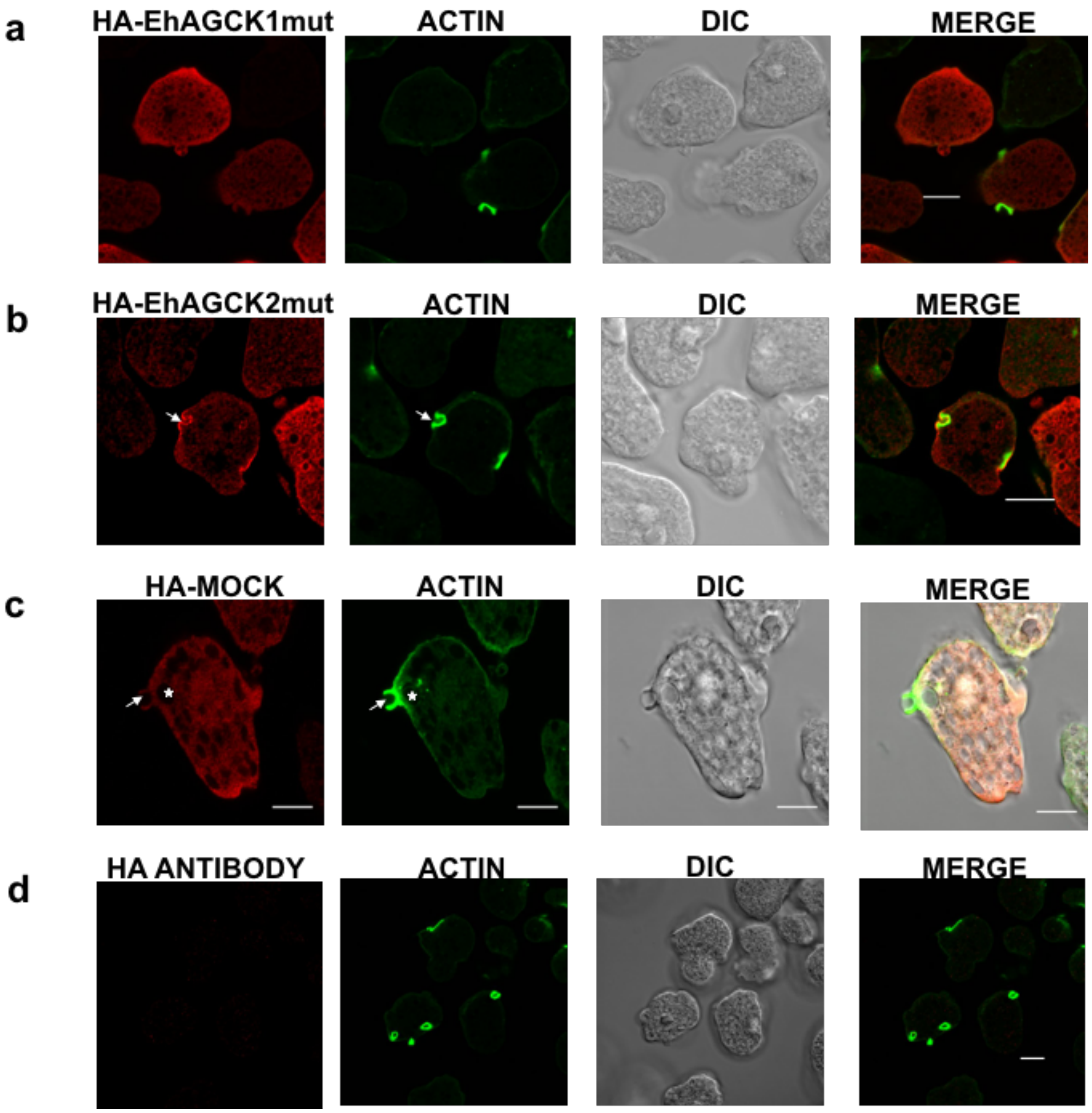

Supplementary Fig. 8. Immunolocalization of (a) HA-EhAGCK1mut and (b) EhAGCK2mut in fixed amoebic cells. The localization pattern is similar to the wild type protein. HA-EhAGCK2mut localizes to the actin rich endocytic structures. (c) Immunolocalization of HA-tagged BAR domain containing cytosolic protein in *E. histolytica* trophozoites. The arrow indicates the actin rich phagocytic cup and star indicates the newly formed phagosome. (d) *E. histolytica* cells transfected with HA vector stained by HA antibody for control experiment. The cells were double labeled with Alexa 546 (EhAGCK1 or 2) and FITC Phalloidin (actin). (Scale bar, 10μm; DIC, Differential Interference Contrast)

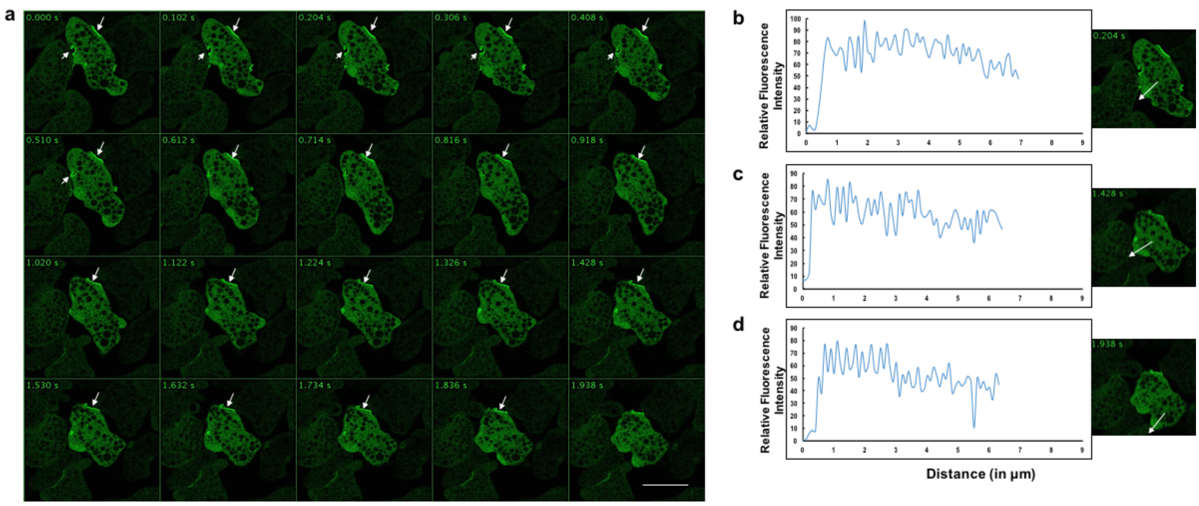

Supplementary Fig. 9. (a) The montage of GFP-EhAGCK2 expressing motile trophozoites. The site on the membrane enriched with GFP-EhAGCK2 have been marked with arrow. (b-d) The pseudopods in three different time frames have been analyzed for GFP-EhAGCK2 intensity along the marked arrow line. The graph shows intensity of GFP-EhAGCK2 in pseudopods versus cytoplasm and plasma membrane. Scale bar, 10μm.

a

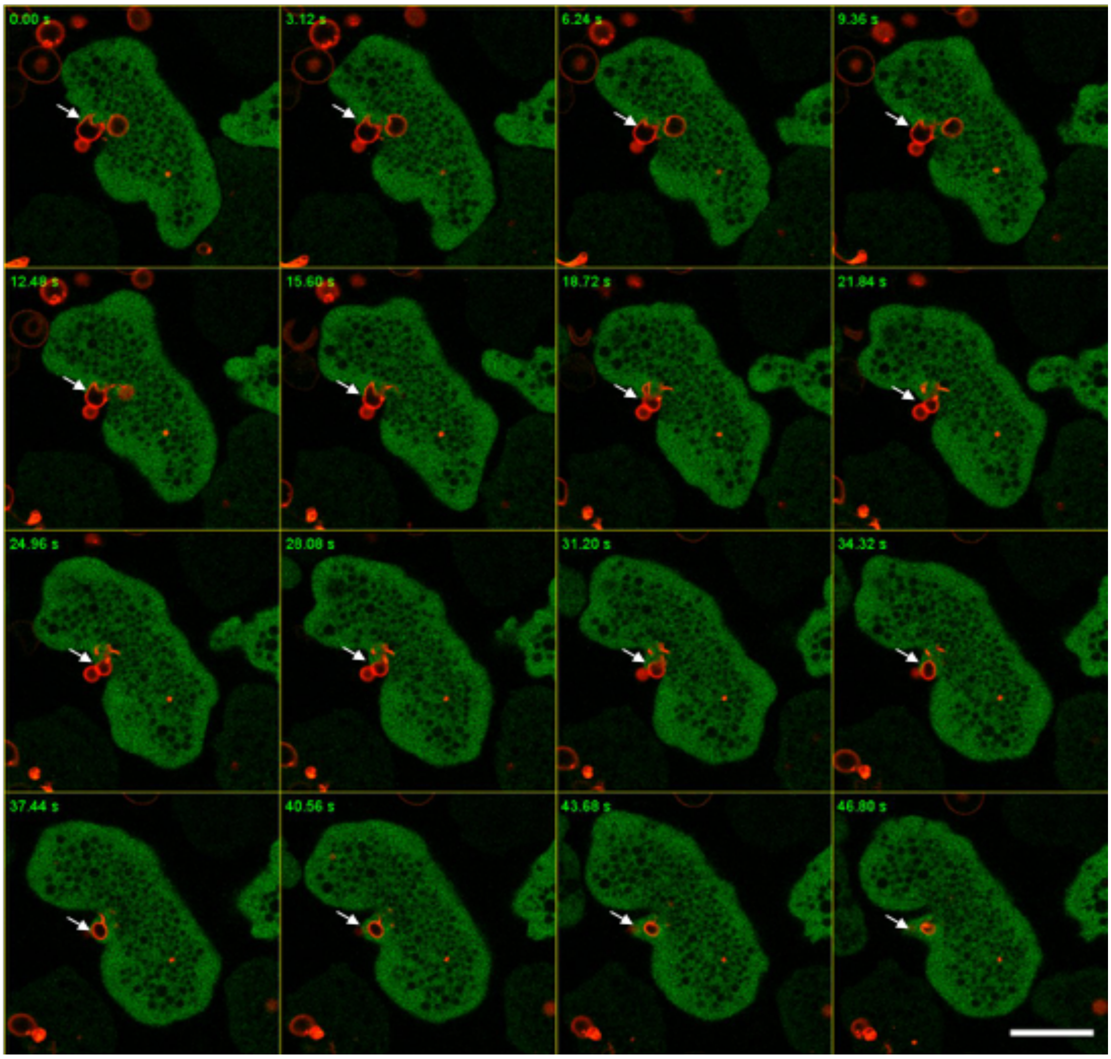
0.00 s
3.12 s
6.24 s
9.36 s
12.48 s
15.60 s
18.72 s
21.84 s
24.96 s
28.08 s
31.20 s
34.32 s
37.44 s
40.56 s
43.68 s
46.80 s

b

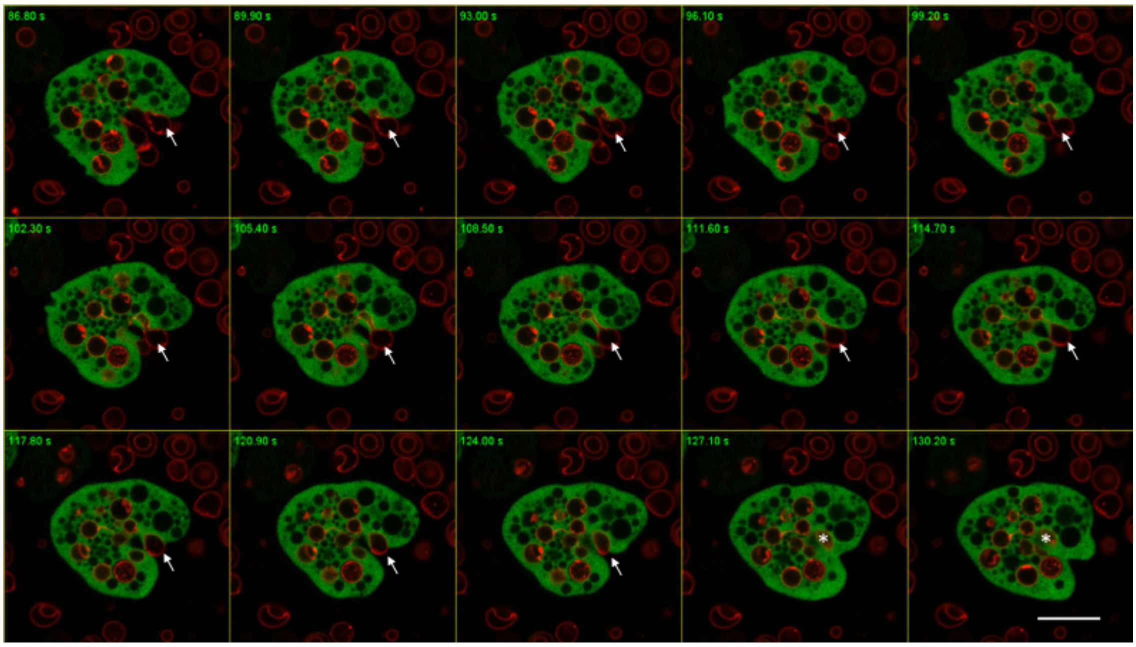
86.80 s
89.90 s
93.00 s
96.10 s
99.20 s
102.30 s
105.40 s
108.50 s
111.60 s
114.70 s
117.80 s
120.90 s
124.00 s
127.10 s
130.20 s

Supplementary Fig. 10. (a) and (b) Time-series montages showing no preferential localization of GFP-EhAGCK1 at the site of erythrophagocytosis in trophozoites expressing GFP-tagged protein. These two montages show consistently that EhAGCK1 is not involved in erythrophagocytosis. The site of erythrophagocytosis is marked by an arrow. Scale bar, 10μm.

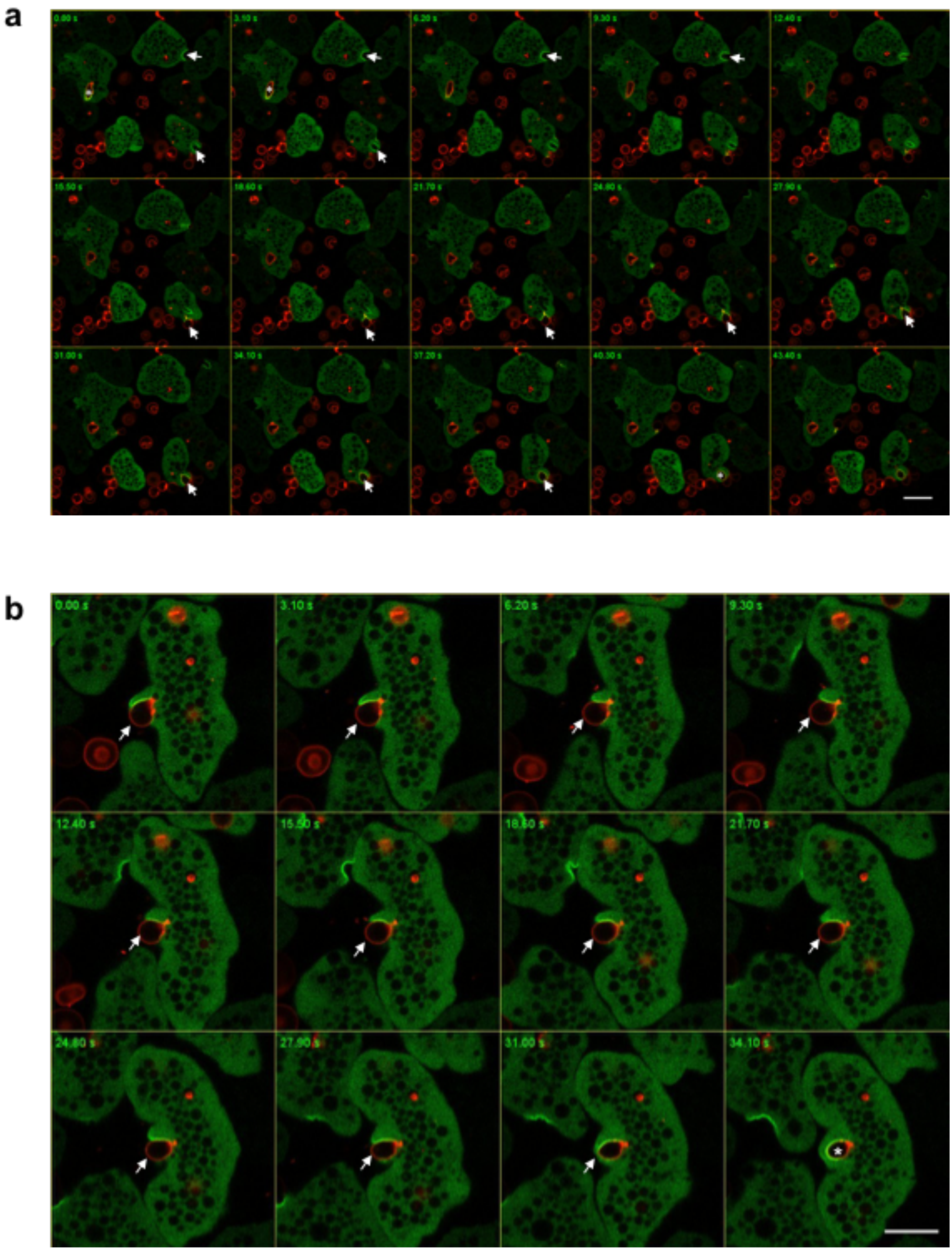

Supplementary Fig. 11. (a) Time-series montage showing recruitment of GFP-EhAGCK2 at multiple events of erythrophagocytosis. (b) Trophozoite expressing GFP-EhAGCK2 show recruitment of tagged protein at the site of erythrophagocytosis in a consistent manner. The newly formed phagosomes are

marked by asterisks and site of phagocytosis is shown by an arrow. Scale bar, 10μm.

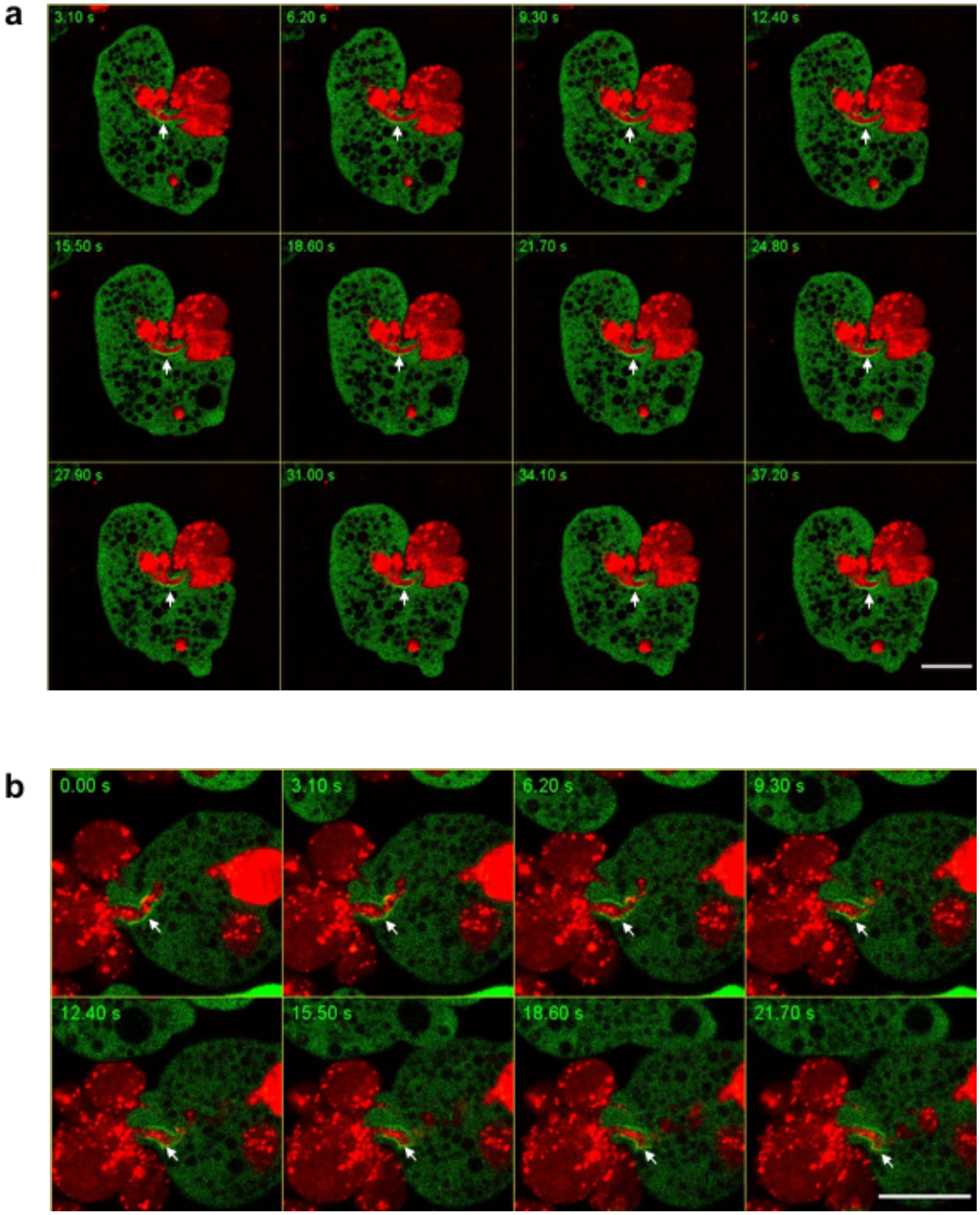

Supplementary Fig. 12. (a) and (b) Trophozoites expressing GFP-EhAGCK1 show recruitment in the tunnel formed during trogocytosis of labeled live CHO cells. The arrows show the site of trogocytosis and tunnel formed during the process. Scale bar, 10μm.

a

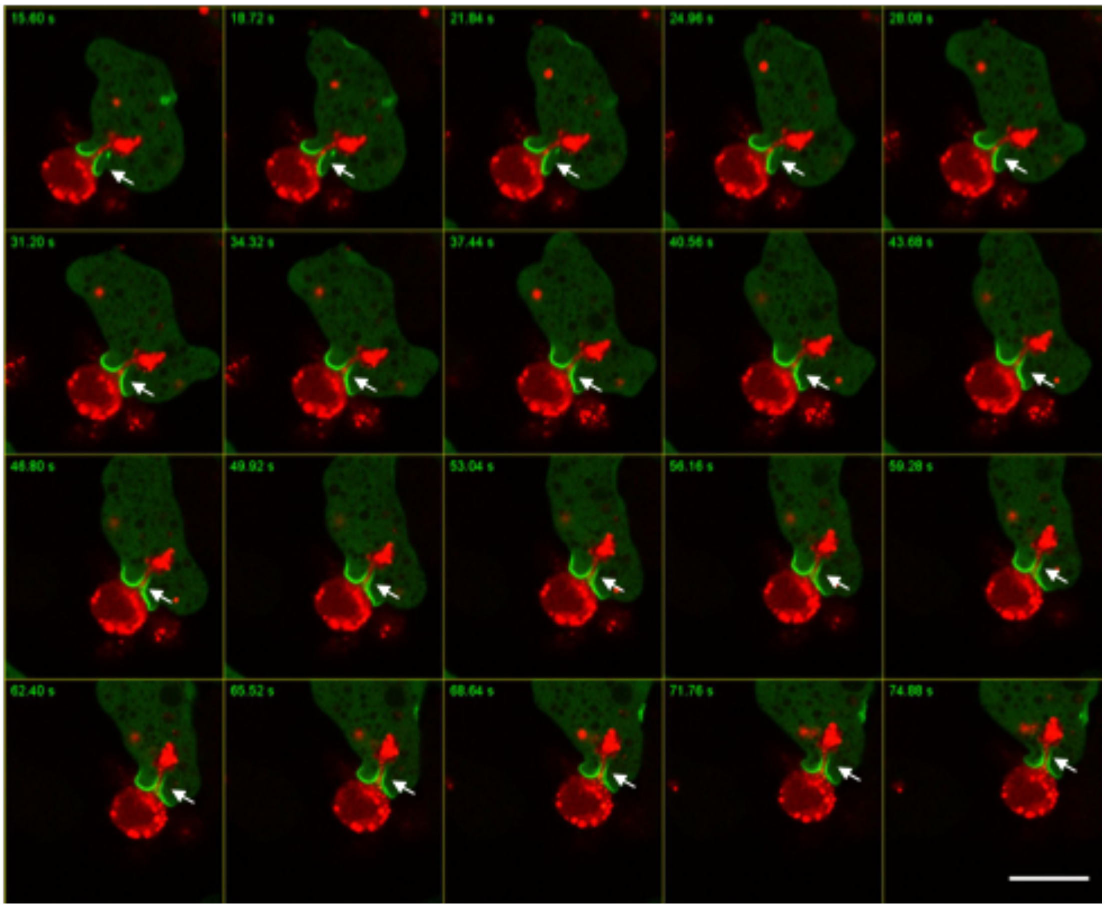
15.60 s
18.72 s
21.84 s
24.96 s
28.08 s
31.20 s
34.32 s
37.44 s
40.56 s
43.68 s
46.80 s
49.92 s
53.04 s
56.16 s
59.28 s
62.40 s
65.52 s
68.64 s
71.76 s
74.88 s

b

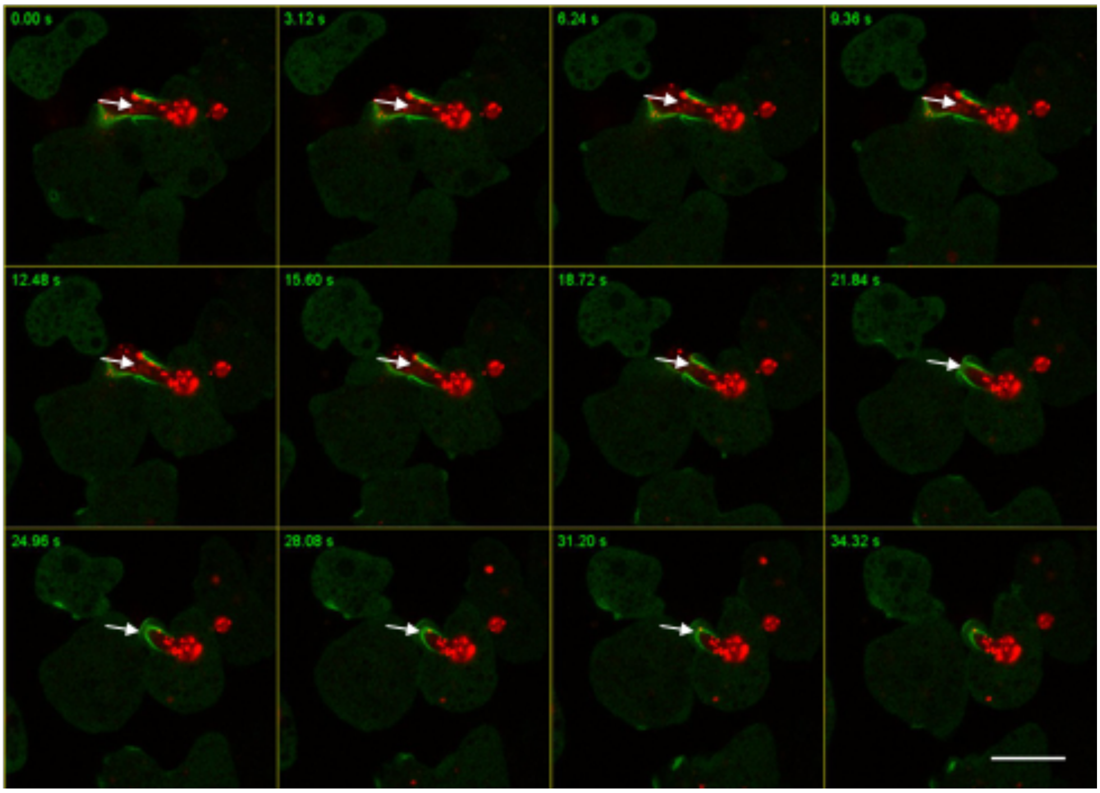

Supplementary Fig. 13. (a) and (b) Time-series montages showing recruitment of GFP-EhAGCK2 at the site of contact with labeled live CHO cells and in the tunnel formed during trogocytosis. The site of trogocytosis is marked by an arrow in the montage. Scale bar, 10μm.

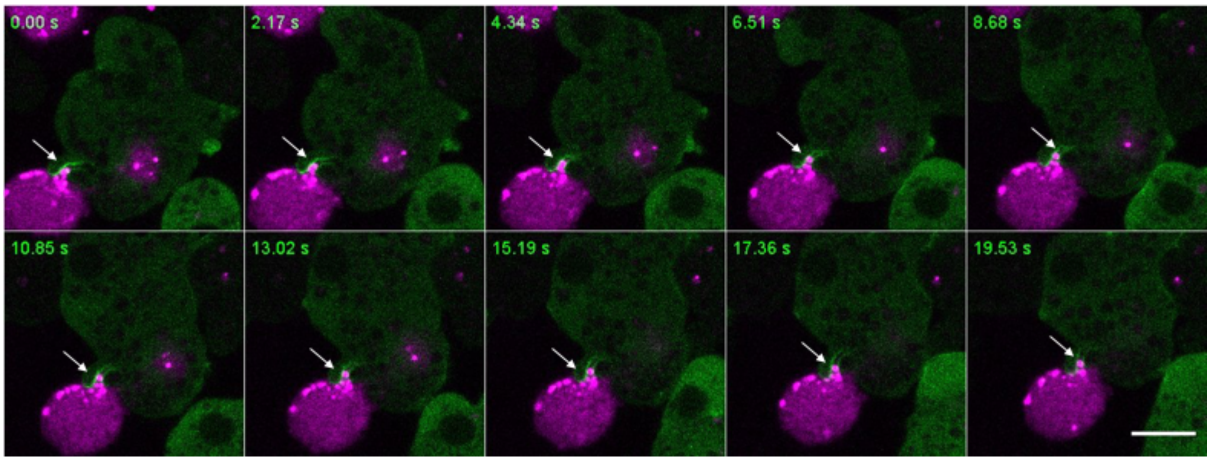

Supplementary Fig. 14. Time-series montage of showing localization of GFP-EhAGCK1 to the tunnel formed during trogocytosis of live Caco-2 cell (marked with arrow). Scale bar, 10μm.

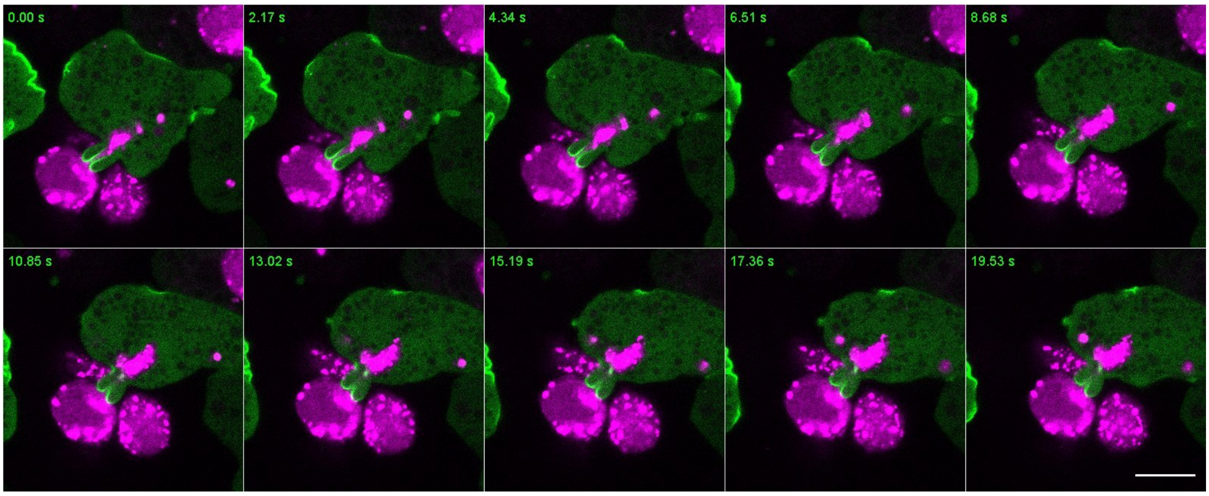

Supplementary Fig. 15. Montage of time series showing localization of GFP-EhAGCK2 to the plasma membrane during trogocytosis of labeled live Caco-2 cell (marked with arrow). Scale bar, 10μm.

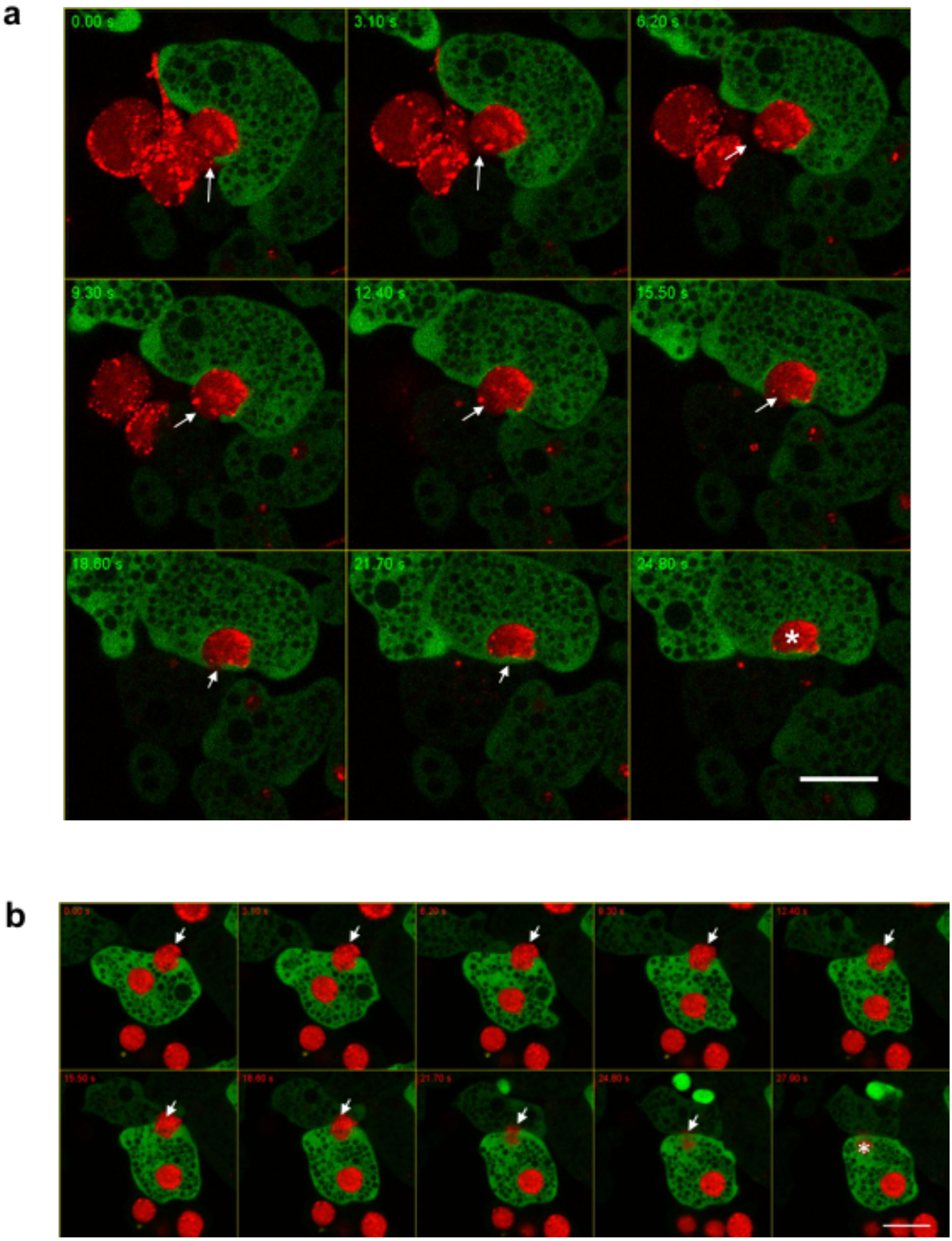

Supplementary Fig. 16. (a) and (b) Time-series montages showing phagocytosis of labeled pre-killed CHO cells by trophozoites expressing GFP-EhAGCK1. Both the montages show no specific recruitment of GFP-EhAGCK1 to the site of

phagocytosis. The site of phagocytosis is marked by an arrow while the newly formed phagosome is shown by asterisk. Scale bar, 10μm.

a

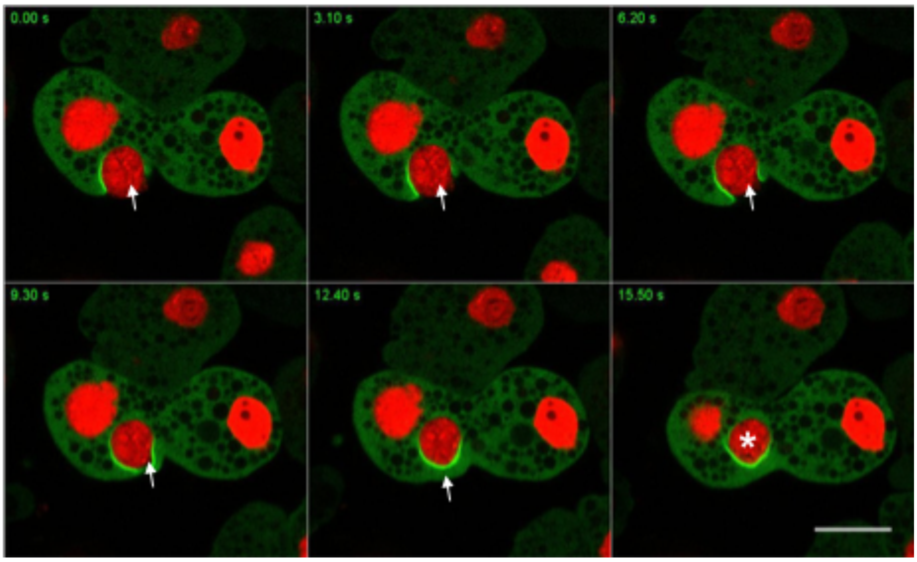

b

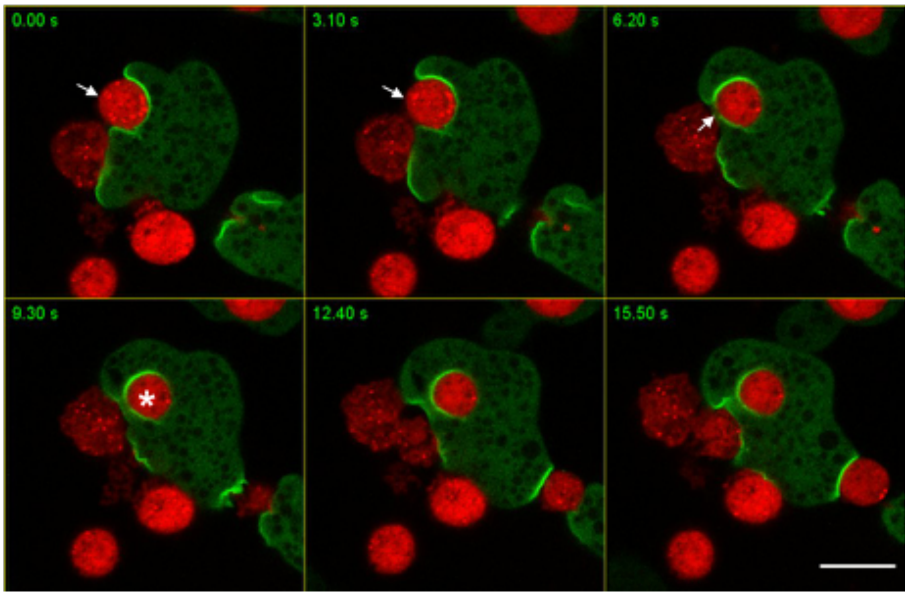

Supplementary Fig. 17. (a) and (b) Live imaging montages showing recruitment of GFP-EhAGCK2 during phagocytosis of pre-killed CHO cells by the trophozoites. The phagocytic cups are indicated by an arrow and newly formed phagosome has been marked by asterisk. Scale bar, 10μm.

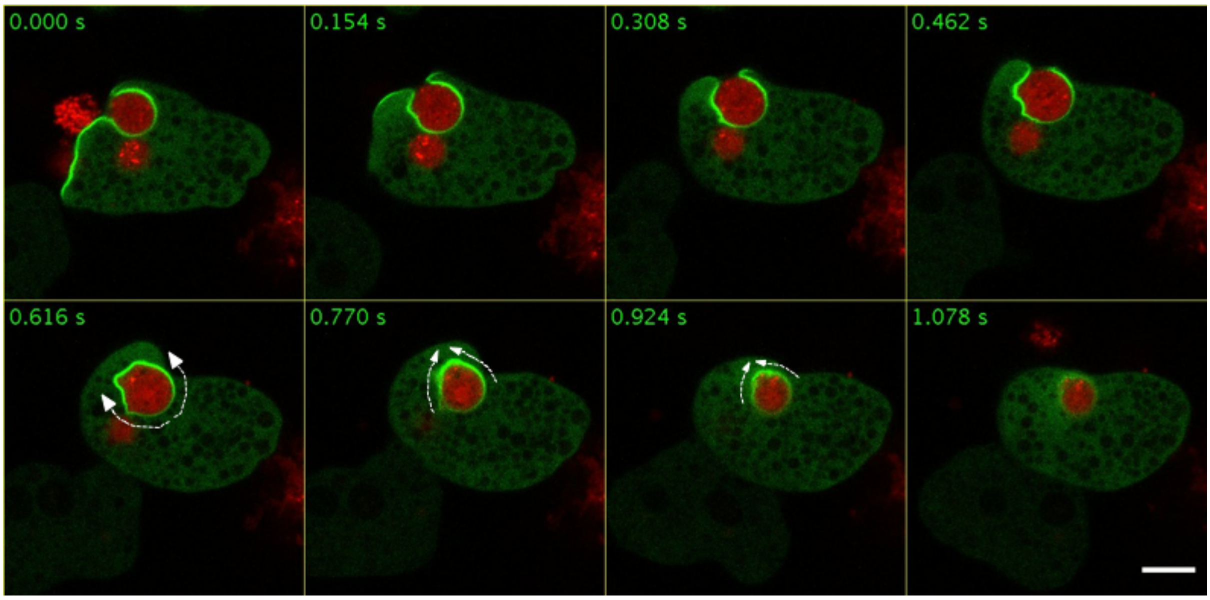

Supplementary Fig. 18. Montage showing time series of trophozoite expressing GFP-EhAGCK2 engulfing labelled pre-killed CHO cell through phagocytosis. The direction of disassociation of GFP-EhAGCK2 around newly formed phagosome is marked by dotted arrow line. Scale bar, 10μm.

Supplementary Table 1. Results of mass spectrometry data obtained for profiling of various forms of phosphatidylinositol binding proteins.

	PtdIns(3,4,5)P_3 Binding protein		PtdIns(4,5)P_2 Binding protein		PtdIns(3)P Binding protein	
1	Acc. No.	% coverage	Acc. No.	% coverage	Acc. No.	% coverage
2	EHI_095940	61	EHI_105110	20	EHI_060740	16
3	EHI_188930	46	EHI_164400	18	EHI_042170	7.8
4	EHI_008090	42	EHI_005970	12	EHI_152050	5.4
5	EHI_040420	31	EHI_001050	10	EHI_167300	5.1
6	EHI_042150	30	EHI_153340	5.7	EHI_092080	3.7
7	EHI_197790	27	EHI_131050	3.8	EHI_076870	3.2
8	EHI_053040	25	EHI_143590	3.4	EHI_198760	3.1
9	EHI_105110	21	EHI_093750	2.8		
10	EHI_010510	17				
11	EHI_125820	17				
12	EHI_103720	15				
13	EHI_138540	14				
14	EHI_091510	11				
15	EHI_009390	8.7				
16	EHI_127250	7.1				
17	EHI_097620	6.4				
18	EHI_156210	5.1				
19	EHI_192460	3.1				

Supplementary Table 2. Sequences of the primers used for amplification of EhAGCK1 and 2 genes.

S.No	Primer Name	Primer Sequence
1.	EhAGCK1F	5’ CCGCCCGGGATGGAAGAGAAAACACAAGGATGGC 3’
2.	EhAGCK1R	5’ CCGCTCGAGTTATTTGCAATAAGTAAAATCATTGAAATAATC 3’
3.	EhAGCK2F	5’ CCGCCCGGGATGTCAGTTAAAACAGGATGGGG 3’
4.	EhAGCK2R	5’ CCGCTCGAGTTATTTTTGTACGTAAGTAAAATCTG 3’
